# Neurologic sequelae of severe chikungunya infection in the first 6 months of life: a prospective cohort study 24-months post-infection

**DOI:** 10.1186/s12879-021-05876-4

**Published:** 2021-02-16

**Authors:** Roelof van Ewijk, Minke H. W. Huibers, Meindert E. Manshande, Ginette M. Ecury-Goossen, Ashley J. Duits, Job C. Calis, Aleid G. van Wassenaer-Leemhuis

**Affiliations:** 1 Saint Elisabeth Hospital, Willemstad, Curaçao; 2 Emma Children’s Hospital, Amsterdam UMC, University of Amsterdam, Amsterdam, the Netherlands; 3 Princess Máxima Center for Pediatric Oncology, Heidelberglaan 25, 3584 CS Utrecht, the Netherlands; 4 Global Child Health Group, Amsterdam UMC, University of Amsterdam, Amsterdam, the Netherlands; 5 Red Cross Blood Bank Foundation Curaçao, Willemstad, Curaçao

**Keywords:** Chikungunya virus, Neurologic manifestations, Child development, Alphavirus infections, Infant, Infant, Newborn, diseases

## Abstract

**Background:**

Perinatally chikungunya infected neonates have been reported to have high rates of post-infection neurologic sequelae, mainly cognitive problems. In older children and adults chikungunya does not appear to have sequelae, but data on postnatally infected infants are lacking.

**Methods:**

We performed a prospective, non-controlled, observational study of infants infected before the age of 6 months with a severe chikungunya infection during the 2014–2015 epidemic in Curaçao, Dutch Antilles. Two years post-infection cognitive and motor - (BSID-III) and social emotional assessments (ITSEA) were performed.

**Results:**

Of twenty-two infected infants, two died and two were lost to follow up. Eighteen children were seen at follow-up and included in the current study. Of these, 13 (72%) had abnormal scores on the BSID-III (cognitive/motor) or ITSEA.

**Conclusion:**

In the first study aimed at postnatally infected infants, using an uncontrolled design, we observed a very high percentage of developmental problems. Further studies are needed to assess causality, however until these data are available preventive measure during outbreaks should also include young infants. Those that have been infected in early infancy should receive follow up.

**Supplementary Information:**

The online version contains supplementary material available at 10.1186/s12879-021-05876-4.

## Introduction

Chikungunya virus is a mosquito-borne alphavirus, known for causing acute emerging public health problems. A small group of patients presents with severe disease. Infants, immunocompromised patients and the elderly are at higher risk for severe disease, with neuro-invasive manifestations and multi-organ involvement. Recent studies have described an association between chikungunya and neurological sequelae [1–3].

Since 2000, large outbreaks of the virus have spread to previously non-endemic regions. In addition the chikungunya virus, which is normally transmitted by *Aedes aegypti* has adapted genetically to *Aedes albopictus*, posing a threat for a more global distribution of the virus [4–7]. In 2013, the first cases of an outbreak in the Western hemisphere of the Asian genotype of the virus were identified. From there, more than a million confirmed or suspected cases were identified in 48 countries or territories in the Caribbean and the Americas [4]. On the island of Curaçao, part of the Dutch Antilles, 835 laboratory proven cases of chikungunya virus infections, of the Asian genotype were reported. It is estimated that 50.000–75.000 inhabitants were infected, corresponding to 30–50% of the total population [2, 8].

Patients with chikungunya infections generally present with high fever, arthralgia, and an erythematous, maculopapular rash, and irritability in infants specifically. Severe infection can present with encephalitis, myocarditis, hepatitis, and multi-organ failure [4, 9]. Convulsions, encephalopathy and intracranial haemorrhage, in some cases leading to death, have been reported [1, 10–14]. Infections occur either through vertical transmission from infected women to newborns peri-partum or through mosquito bites [1, 13]. Information about long-term cognitive effects among infected infants is scarce. Available data show suggest significant global cognitive delay in 51% of mother-to-child vertical infected neonates at 2 years of age [3].

During the chikungunya epidemic in Curaçao in 2014–2015, infants under the age of 6 months with suspected chikungunya infection were admitted to hospital for clinical observation and analysis according to local protocol. We observed severe disease in perinatal and postnatal infected infants [10, 15]. We hypothesized that young infants who had been postnatally infected in the first 6 months of life, could be prone to global developmental delay as well. The objective of this study was to evaluate whether these chikungunya infected neonates and infants presenting with severe disease were prone to developmental deficits.

## Methods

A prospective cohort study was conducted between January 2017 and April 2017. Participants in this study were selected from our previously described cohorts [10, 15]. Baseline data was collected retrospectively. The follow-up study was developed prospectively. The research protocol was developed according to the RECORD guidelines (Reporting of studies Conducted using Observational Routinely-collected health Data) and the ICMJE recommendations (Recommendations for the conduct, reporting, editing, and publication of scholarly work in medical journals) [16, 17].

The study was approved by the Medical Ethical Board of the Saint Elisabeth Hospital in Curaçao (reference number 2016–002). All parents of the children who entered this study signed a written informed consent. Documents and informed consent were provided in both official languages, Dutch and Papiamentu.

### Case definition

Infants younger than 6 months of age who presented during the peak of the chikungunya epidemic (2014–2015) with typical clinical symptoms of chikungunya (fever, maculopapular rash, irritability), neurologic symptoms and/or sepsis-like illness and a laboratory proven chikungunya infection, as defined by the WHO, were included [18]. Infants were admitted to the Saint Elisabeth Hospital, which is the only hospital on the island equipped with a level IIIB neonatal intensive care unit. Diagnosis of chikungunya was confirmed through serum-specific IgM and IgG antibodies by ELISA technique (Euroimmune AG, Lubeck, Germany), WHO cut-offs. To identify mother-to-child transmission, samples were taken from infants and mothers during initial infection and during short-term follow-up (1–4 months). Diagnosis was made by IgM or a 4-fold IgG increase with at least 14 days between measurements [18]. Perinatal infections (mother-to-child transmission) were defined as cases with confirmed infection in mother and neonate as well as clinical symptoms of chikungunya before the 8th day of life [4, 5].

Neurologic symptoms were defined as profound irritability/neurologic agitation (identified by clinical observation), encephalitis, encephalopathy, convulsions or intracranial haemorrhage. Sepsis-like illness was defined as having 2 or more systemic inflammatory response syndrome (SIRS) criteria, which are temperature > 38.5 °C or < 36 °C; heart rate > 2SD for age; respiratory rate > 2SD for age; WBC elevated or depressed for age, or > 10% immature neutrophils [10, 15].

#### Medical history

All children were evaluated by retrospective file review and parental report for no hospital admissions, convulsions, traumatic brain injury, illnesses, medication use or other significant medical history affecting development. Maternal records were retrospectively screened to document maternal illnesses, periods of fever, screening results of cognitive imperative infections (Toxoplasma, Rubella, CMV, Herpes, Syphilis - TORCHES) to assess potential confounders.

#### Follow-up

Development was assessed around the second birthday using the Bayley Scales for Infant Development 3rd edition (BSID-III, US edition). Children were seen by a paediatrician to evaluate medical history, current medication and demographic situation. Standardized physical and neurologic examination were performed. If acute conditions were identified that could possibly interfere with the BSID-III assessment, it was postponed. The BSID-III motor evaluation and Amiel-Tison muscle tone score were performed by a paediatric physiotherapist. The BSID-III cognitive evaluation was performed by a paediatric psychologist. Both of these examiners were previously schooled and familiar with the BSID-III. The social emotional development was assessed by the Infant Toddler Social Emotional Assessment parent questionnaire (ITSEA). All investigations were performed by the same team. The team was aware of the previous chikungunya infection, but unaware of details of the medical history.

### Outcome measures at follow-up

The primary outcome measures were epilepsy, abnormal neurologic examination, BSID-III cognitive and motor composite score, Amiel-Tison muscle tone score and ITSEA T-scores for the internalizing, externalizing, dysregulating and competence domain.

The BSID-III assesses the developmental level for children between 16 days and 42 months. It generates 3 composite scores for cognitive, motor and language, as well as 5 subsets adjusted to age and sex [19, 20]. All parents spoke Dutch, but the home language of most children was the local language (Papiamentu). Therefore, language scores were omitted. Cognitive, motor composite, scaled fine motor and scaled gross motor scores were used to evaluate developmental outcome. The BSID-III has a mean of 100. Cut-off points for mild/moderate and moderate delay were <  85 and < 70, (< 1 and < 2 standard deviation, respectively). All test administrators were fluent in Papiamentu. If the child’s main language was Papiamentu, the BSID-III was administered that language.

The ITSEA is a 169-item, parent-reported questionnaire for evaluation of social emotional problems and competencies in 12- to 36-month old children. We used the validated Dutch language version [21, 22]. The value of the ITSEA is that it systematically evaluates for a wide range of problem behaviours and competencies, revealing problems that often co-occur with the core symptoms of psychiatric disorders. The main three problem domains are internalizing, externalizing and the dysregulation domain. There is also a competence domain. T-scores are calculated based on age and sex with a mean score of 50 and a standard deviation of 10 (range 25–80). Abnormal scores on the problem and competence domains are ≥65 (≥1.5SD) and on the competence domain a score of ≤35 (≤1.5SD), respectively. The ITSEA has an acceptable test-retest reliability [22–24].

The *Amiel-Tison muscle tone score*, which is part of the Amiel-Tison neurologic assessment, is a standardized way to assess the child’s active and passive muscle tone. We categorized children in hypotonic, hypertonic or normal muscle tone [25].

The levels of parental education and parental employment were used to measure the socioeconomic status. Parental education was assessed by the European Survey Version of the International Standard Classification of Education (ES-ISCED) [26]. Levels of education were categorized as ‘low level of education’ (lowest type of college or less; total years post-elementary schooling < 6), ‘middle level of education’ (middle-level college; total years post-elementary schooling 6–8) or ‘high level of education’ (highest-level college or university; total years post-elementary schooling > 8). The combined parental education score was low if one or both parents had a low level of education, middle if both parents had a middle-level education or high – low education, and high if one or both parents had a high-level education [27].

### Data analysis

Data were entered in SPSS (IBM Version 24, 2016). Means and standard deviations were calculated for BSID-III composite scores, motor scaled scores and ITSEA T-scores. Subgroup analysis was performed to explore whether outcomes were influenced by measures of disease severity or socio-demographic background variables: BSID-III/ITSEA for sex (male - female), type of infection (perinatal - postnatal), combined parental education (high - middle - low), household situation (single-parent - family), household smoking (yes - none), gestational age (preterm - term) and duration of admission (< 7 days – ≥7 days) were calculated. For each possible confounder Levene’s test for equality of variances and t-test for equality of means were performed with a significance level of *p* < 0.05. For children with a BSID-III cognitive score <  85, ANOVA and Fischer’s exact tests were performed to identify associations.

## Results

### Population characteristics

Of the 27 infants enrolled in our previous cohorts [10, 15], 22 had neurologic symptoms or sepsis-like illness and were thereby eligible for this study. Maternal records did not identify other infectious diseases (TORCHES) during pregnancy. Two neonates, both with perinatal infections, died during their initial chikungunya infection due to previously chikungunya-associated complications; one due to persistent status epilepticus, the other due to intracranial haemorrhage. Two other children were lost to follow-up (*n* = 1) or did not consent to enrolment (*n* = 1). Thus, 18 of 22 eligible children were enrolled in study. Of these 18 infants, 3 were infected through mother-to-child vertical transmission and 15 in the postnatal phase. Baseline details are presented in Table 1. Individual baseline and laboratory details are presented in Table S1. Three preterm infants were all moderate to late preterm with a gestational age of 34 5/7, 35 0/7 and 36 5/7 weeks respectively. They all had postnatal chikungunya infection.

**Table 1:** Baseline characteristics and sociodemographic characteristics

***Total number of children (N = 18)***	N	%
Gender: female	11	61.1
Perinatal infection	3	16.7
Postnatal infection	15	83.3
Preterm < 37 weeks	3	16.7
*Admission*	Mean	SD
Age at admission (days)	64.8	53.9
Duration of admission (days)	6.2	3.2
*Age at admission*	N	%
< 1 month	5	27.8
1–3 months	8	44.4
4–6 months	5	27.8
*Symptoms*		
Profound irritability	17	94.4
Convulsions	1	5.6
Fever	17	94.4
Rash	12	66.7
*Laboratory*		
Leukopenia (< 6 x 10E9/L)	7	38.9
Leukocytosis (> 15 x 10E9/L)	2	11.1
Thrombocytopenia (< 100 x 10E9/L)	1	5.6
Increased CRP (> 15 mg/L)	12	66.7
*Treatment*		
Antibiotics	15	83.3
*Assessment*	Mean	SD
Age (months)	28	2.17
*Sociodemographic determinants*		
*Maternal ethnicity*		
Afro-Caribbean	14	77.8
Asian	1	5.6
Caucasian	1	5.6
Latin American	2	11.1
*Paternal ethnicity*		
Afro-Caribbean	14	77.8
Caucasian	3	16.7
Latin American	1	5.6
*Combined parental education*		
Low level	10	55.6
Middle level	5	27.8
High level	3	16.7
*Mother: paid job*		
Employed and paid	15	83.3
Taking care of household	1	5.6
Unemployed	2	11.1
*Father: paid job*		
Employed and paid	13	72.2
Unemployed	1	5.6
Unknown	4	22.2
*Household smoking*		
No	12	66.7

Data are total number (N), percentage (%), mean and standard deviation (SD) where eligible.*CRP* C-reactive protein

### Medical history since chikungunya infection

In the period between initial chikungunya infection and follow-up, no convulsions, traumatic brain injury, hospital admissions, illnesses, medication use or medical history affecting development were reported. One child who had convulsions initially was treated for 3 months with phenobarbital, which was then stopped without recurrence of convulsions. Two parents (11.1%) had concern about possible delay in the development of their children, this mainly focused on language development. Among the entire cohort, the mean age for walking without support was 11 months (SD 2.4).

#### Cognitive development

The mean BSID-III cognitive score was 84.71 (*N* = 17; SD7.4). Seven children had cognitive delay with a composite cognitive score < 85 (N = 17; 41.2%). None had a cognitive composite score ≤ 70 (Table 2). There was no association between cognitive delay and either socioeconomic background and disease severity (Table 3).

**Table 2:** Results: Cognitive assessment by BSID-III and the ITSEA

**BSID-III score**	**Mean**	**SD**	**Median**	**Score < 85**	**%**	**Score ≤ 70**	**%**
Motor	106.8	15.6	105	1	5.6	0	0.0
Cognitive (N = 17)	84.7	7.4	85	7	41.2	0	0.0
Gross motor scaled score	10.9	3.6	10.0				
Fine motor scaled score	11.3	2.7	11.0				
**ITSEA T score**	**Mean**	**SD**	**Median**	**Score > 65**	**%**		
Internalizing	56.8	10.9	57	5	27.8		
Externalizing	58.7	12.5	60	5	27.8		
Dysregulation	51.8	11.0	53	3	16.7		
	**Mean**	**SD**	**Median**	**Score < 35**	**%**		
Competence	50.1	11.0	50	2	11.1		
				**N**	**%**		
≥1 abnormal T score				11	61.1		
≥1 abnormal T or BSID-III score				13	72.2		

All included infants [18] were assessed. One child’s BSID-III cognitive score could not be assessed. Data are presented in number (N), percentage (%), standard deviation (SD) and median

**Table 3:** Results: BSID-III and ITSEA explorative analysis based on combined parental education level and infection type

	Combined parental education						Infection type			
	Low		Middle		High		Perinatal		Postnatal	
BSID-III score	*Mean*	*SD*	*Mean*	*SD*	*Mean*	*SD*	*Mean*	*SD*	*Mean*	*SD*
Number of infants	10		5		3		3		15	
Motor	105.3	14.1	100.8	16.9	122.0	12.5	100.0	13.8	108.2	16.0
Score < 85 (%)	0.0		20.0		0.0		0.0		6.7	
Number of infants	9		5		3		3		14	
Cognitive	83.3	6.1	83.0	9.1	91.7	5.8	88.3	2.9	83.9	7.9
Score < 85 (%)	44.4		60.0		0.0		0.0		50.0	
ITSEA (T score)	*Mean*	*SD*	*Mean*	*SD*	*Mean*	*SD*	*Mean*	*SD*	*Mean*	*SD*
Number of infants	10		5		3		3		15	
Internalizing	60.9	10.7	52.0	7.8	51.3	13.7	67.7	4.7	54.7	10.5
Externalizing	63.4	11.0	54.4	12.5	50.3	14.6	58.0	19.9	58.9	11.6
Dysregulation	56.9	9.4	50.2	8.8	37.7	6.4	56.3	20.2	50.9	9.1
Competence	51.6	12.5	48.6	11.4	47.7	6.5	60.7	5.8	48.0	10.7

Data are presented in mean or standard deviation (SD)

Children with a BSID-III cognitive score < 85 had similar mean motor and ITSEA domain scores as children with a cognitive score ≥ 85 (Table 4).

**Table 4:** T-test for equality of means for BSID-III and ITSEA for cognitively delayed children

BSID-III cognitive score	< 85		≥ 85		T test
Total number of infants	7		10		
**BSID-III score**	**Mean**	**SD**	**Mean**	**SD**	**P**
Motor	102.6	18.4	107.8	13.5	0.504
Motor scaled fine motor	10.3	2.6	11.7	2.6	0.283
Motor scaled gross motor	10.6	4.4	10.8	3.2	0.915
**ITSEA T score**					
Internalizing	58.1	6.6	54.2	12.4	0.409
Externalizing	61.7	13.2	56.1	12.8	0.392
Dysregulation	54.4	9.3	49.9	12.7	0.435
Competence	47.3	8.6	50.5	12.1	0.556

Data are presented in number, mean and standard deviation (SD)

#### Motor development

One child had mild to moderate motor delay (5.6%) (Table 2). None of the children showed abnormalities at neurologic examination. All children (N = 18) had normal Amiel-Tison muscle tone scores. There were no risk factors that were significantly associated with motor outcome.

#### Social emotional development

Eleven children (61.1%) had abnormal ITSEA scores, with internalizing and externalizing domains mainly affected. The dysregulation domain was significantly higher in the low parental education group, as compared with the middle/high parental education group (*p* = 0.007). In preterm infants (*n* = 3) the internalizing scores were significantly higher (mean 66.67; SD 2.08) compared to term infants (mean 54.87; SD 54.9) (*p* = 0.001).

## Discussion

This is the first study to describe long-term neurological outcomes in a highly selected cohort of infants with a severe chikungunya infection in their first 6 months of life. Using an uncontrolled design, we identified delayed cognitive development in up to 41.2% of the infants, and an abnormal social emotional development score (ITSEA) in 61% of the infants presenting with a severe chikungunya infection. In as much as 72% of the children we found an abnormal score on either BSID-III or ITSEA. The delayed cognitive development was not attributable to the included perinatally infected children. We did not observe other sequelae like epilepsy or abnormal muscle tone. There was a mix of socio-emotional problems.

Recent evidence describes long-term sequelae associated with chikungunya virus infections in vulnerable patients. A systematic review of long-term sequelae described three studies focussing on mother-to-child transmission [3, 13, 28], of which the CHIMERE cohort study of Gérardin et al. (2014) was the only one that addressed development after perinatal chikungunya infection. Our study is the first to investigate child development after a postnatal chikungunya infection [3]. In the CHIMERE cohort, severe illness was seen in 52.6% of the vertically infected neonates, of which nine developed encephalopathy and two intracranial haemorrhage [1, 13]. The CHIMERE cohort study (*N* = 33) showed that 51% of the vertically infected infants had moderate or severe global developmental delay at 2 years of age, as assessed by the Revised Brunet-Lézine (RBL) scale, compared to a control group of 135 uninfected children, wherein 16% had moderate global developmental delay (*p* < 0.001). When these authors also included subscores, 73.9% of the infected infants had a developmental deficit. The non-controlled design and inclusion of a different population limits detailed comparison with our infants. Nevertheless, these figures are in the same range as our results with cognitive delay in 41.2% and any abnormal score in up to 72.2%, but there were no children with severe delays (cognitive score < 70) in our case series. The CHIMERE cohort study combined children with encephalitis and ‘mild prostration’ (defined as neonates unable to be breastfed or bottle-fed), and even in this ‘mild prostration’ group, up to 38.1% had a global developmental deficit, underlining the seriousness of the neuroinvasive effects of chikungunya to the young brain [1, 3]. Although arthritis and chronic arthralgia are the most frequent long-term sequelae in adults, in our cohort, similarly to the CHIMERE cohort, the developmental delay was mainly cognitive and the motor development was less affected [1, 3].

A systematic review of neuroinvasive chikungunya showed a bimodal patient age distribution, with complications more frequently seen in the youngest and the elderly [29]. Among neonates, infants and elderly with comorbidities, neurologic complications, mainly encephalitis and acute encephalopathy, are assumed to be directly related to the acute infection. In contrast, for otherwise healthy adults there is a symptom-free interval between the infection and neurologic disorders (such as acute disseminated encephalomyelitis, optic neuropathy and Guillain–Barré syndrome) suggesting that these neurologic complications are due to an underlying autoimmune process [29]. The neuroinvasive potential of the chikungunya virus is supported by in vitro studies that demonstrate that the virus is capable of infecting mouse brain neurons, astrocytes and oligodendrocytes. The infection induces apoptosis in neurons and astrocytes, together with initializing the production of pro-apoptotic factors [29–31]. This supports our hypothesis that young infants who lack a fully developed, mature innate and adaptive immune response as well as perinatally infected neonates are at risk for developmental disorders, although the exact pathogenesis in humans remains to be determined [3, 29]. Since the chikungunya virus does not cross the placenta [13], we presume that the developmental delay among perinatally infected neonates is induced after birth analogous to postnatally infected young infants. Future research will have to determine up to what age and which infants are at risk for developmental delay.

Although the 2 years outcomes were comparable, the genotype of the chikungunya virus in the CHIMERE cohort on La Réunion was different from that in Curaçao. In Curaçao the Asian genotype was identified whereas the one in the CHIMERE cohort was the East Central South Africa genotype (ECSA) [3, 8]. The neurovirulence of the Asian genotype was recognized with increasing reports of neurologic complications in cases infected in the Americas after the 2014 epidemic. Although in vivo mice studies performed with the ECSA and the Asian genotype demonstrated neurovirulence with both strains. The Asian genotype showed higher mortality and higher apoptosis gene expression [29, 32].

Although prospective recruitment is a strength of our study, we acknowledge several major limitations. This is a small sample of a highly selected group of severely ill children. The diagnosis of chikungunya infection was made by immunoglobulin assay because PCR was not available in our laboratory at the time when patients were enrolled in the study. Consequently, some patients may have had co-infection with other viruses. Bacterial cultures were performed on all patients. Although other arboviruses may present with sepsis-like illness and encephalitis with overlapping clinical cases, no long-term cognitive delay of this level has been described for locally circulating arboviruses, mainly dengue and Zika [33, 34]. Of importance, Zika was not identified on Curaçao until 2016 [35]. Therefore it is possible that the cognitive delay that we observed was not the results of chikungunya infection alone. Another important limitation was the lack of a control group. There are no normative studies for the BSID-III and the ITSEA for the Dutch Antilles, nor did we include a control group. Therefore over estimation, but also underestimation of the describes outcomes cannot be excluded. Finally, since the investigators who performed the neurologic and developmental assessments were not blinded, they may have been biased.

Our data contribute to the scarce existing knowledge regarding the impact on development of chikungunya virus infection in young infants. Although causality and the extent to which chikungunya contributes to cognitive delay and the development in late childhood is still to be determined, we believe that these data will be helpful to policy makers and paediatricians caring for infants up to 6 months of age who had chikungunya infection. Future studies are needed to determine the attributable effect of chikungunya on cognitive delay and development in these infants. When causality of chikungunya and developmental deficits are proven, structured interventions can be implemented, which is of importance for participation in society later in life. We hope that these data will inspire researchers to continue investigating the impact of chikungunya infection on suspected vulnerable groups, as well as to encourage the development and implementation of public health measures and therapeutic options, like vaccines and neutralizing antibodies. Advances such as maternal antenatal vaccination could limit the burden of disease in this vulnerable population [29, 30, 36].

## Conclusion

In the first study to describe the effect of chikungunya infections in early infancy, nearly three quarters of the children had mild cognitive delay or social emotional problems at 24 month follow-up. Although there are important limitations to our study, the association of neurologic sequelae with chikungunya infection in infants is worrisome. Further studies are needed to determine causality. In the meantime preventive measures during outbreaks should also include young infants and those who have been infected in early infancy should receive follow up.

## Supplementary Information

**Additional file 1.**

## Data Availability

The datasets used and/or analyzed during the current study are available from the corresponding author on reasonable request.

## Abbreviations

BSID-III: Bayley Scales for Infant Development, 3rd edition
CRP: C-reactive protein
ELISA: Enzyme-linked ImmunoSorbent Assay
ES-ISCED: European Survey Version of the International Standard Classification of Education
ITSEA: Infant Toddler Social Emotional Assessment parent questionnaire
RECORD: Reporting of studies Conducted using Observational Routinely-collected health Data
SD: Standard deviation
SIRS: Systemic inflammatory response syndrome
WBC: White blood cells
WHO: World Health Organization

## References

[1] van Aalst M, Nelen CM, Goorhuis A, Stijnis C, Grobusch MP (2017). Long-term sequelae of chikungunya virus disease: a systematic review. Travel Med Infect Dis.

[2] Elsinga J, Gerstenbluth I, van der Ploeg S, Halabi Y, Lourents NT, Burgerhof JG (2017). Long-term Chikungunya Sequelae in curacao: burden, determinants, and a novel classification tool. J Infect Dis.

[3] Gerardin P, Samperiz S, Ramful D, Boumahni B, Bintner M, Alessandri JL (2014). Neurocognitive outcome of children exposed to perinatal mother-to-child Chikungunya virus infection: the CHIMERE cohort study on Reunion Island. PLoS Negl Trop Dis.

[4] Burt FJ, Chen W, Miner JJ, Lenschow DJ, Merits A, Schnettler E (2017). Chikungunya virus: an update on the biology and pathogenesis of this emerging pathogen. Lancet Infect Dis.

[5] Weaver SC, Lecuit M (2015). Chikungunya virus and the global spread of a mosquito-borne disease. N Engl J Med.

[6] Thiberville SD, Moyen N, Dupuis-Maguiraga L, Nougairede A, Gould EA, Roques P (2013). Chikungunya fever: epidemiology, clinical syndrome, pathogenesis and therapy. Antivir Res.

[7] Brizzi K (2017). Neurologic manifestation of Chikungunya virus. Curr Infect Dis Rep.

[8] Anfasa F, Provacia L, GeurtsvanKessel C, Wever R, Gerstenbluth I, Osterhaus AD (2017). Hyperferritinemia is a potential marker of chronic chikungunya: a retrospective study on the island of curacao during the 2014-2015 outbreak. J Clin Virol.

[9] Elenga N, Folin M, Vandamme YM, Cuadro-Alvarez E, Long L, Njuieyon F (2017). Chikungunya infection in hospitalized febrile infants younger than 3 months of age. Pediatr Infect Dis J.

[10] van Enter BJD, Huibers MHW, van Rooij L, Steingrover R, van Hensbroek MB, Voigt RR (2018). Perinatal outcomes in vertically infected neonates during a Chikungunya outbreak on the island of curacao. Am J Trop Med Hyg.

[11] Economopoulou A, Dominguez M, Helynck B, Sissoko D, Wichmann O, Quenel P (2009). Atypical Chikungunya virus infections: clinical manifestations, mortality and risk factors for severe disease during the 2005-2006 outbreak on Reunion. Epidemiol Infect.

[12] Gerardin P, Couderc T, Bintner M, Tournebize P, Renouil M, Lemant J (2016). Chikungunya virus-associated encephalitis: a cohort study on La Reunion Island, 2005-2009. Neurology..

[13] Gerardin P, Barau G, Michault A, Bintner M, Randrianaivo H, Choker G (2008). Multidisciplinary prospective study of mother-to-child chikungunya virus infections on the island of La Reunion. PLoS Med.

[14] Almeida Bentes A, Kroon EG, Romanelli RMC (2019). Neurological manifestations of pediatric arboviral infections in the Americas. J Clin Virol.

[15] van Keulen V, Huibers M, Manshande M, van Hensbroek MB, van Rooij L (2018). Chikungunya Virus Infections Among Infants-Who Classification Not Applicable. Pediatr Infect Dis J..

[16] Benchimol EI, Smeeth L, Guttmann A, Harron K, Moher D, Petersen I (2015). The REporting of studies conducted using observational routinely-collected health data (RECORD) statement. PLoS Med.

[17] International Committee of Medical Journal Editors. Recommendations for the Conduct, Reporting, Editing and Publication of Scholarly Work in Medical Journals. Available from: http://www.ICMJE.org. Accessed 1 Jan 2020. 25558501

[18] (Organisation) WWH (2008). Guidelines on Clinical Management of Chikungunya Fever.

[19] Bayley N (2006). Bayley scales of infant and toddler development.

[20] Johnson S, Moore T, Marlow N (2014). Using the Bayley-III to assess neurodevelopmental delay: which cut-off should be used?. Pediatr Res.

[21] Visser JC, Smeekens S, Riksen-Walraven JMA and van Bakel HJA. Infant Toddler Social Emotional Assessment (ITSEA), Dutch Version. Unpublished.

[22] Visser JC, Smeekens S, Rommelse N, Verkes RJ, van der Gaag RJ, Buitelaar JK (2010). Assessment of psychopathology in 2- to 5-year-olds: applying the infant-toddler social emotional assessment. Infant Ment Health J.

[23] Briggs-Gowan MJ, Carter AS (2007). Applying the Infant-Toddler Social & Emotional Assessment (ITSEA) and brief-ITSEA in early intervention. Infant Ment Health J.

[24] Carter AS, Briggs-Gowan MJ, Jones SM, Little TD (2003). The infant-toddler social and emotional assessment (ITSEA): factor structure, reliability, and validity. J Abnorm Child Psychol.

[25] Amiel-Tison C, Stewart A (1989). Follow up studies during the first five years of life: a pervasive assessment of neurological function. Arch Dis Child.

[26] Schneider SL (2010). Nominal comparability is not enough: (in-)equivalence of construct validity of cross-national measures of educational attainment in the European social survey. Res Soc Strat Mobil.

[27] Potharst ES, van Wassenaer AG, Houtzager BA, van Hus JW, Last BF, Kok JH (2011). High incidence of multi-domain disabilities in very preterm children at five years of age. J Pediatr.

[28] Laoprasopwattana K, Suntharasaj T, Petmanee P, Suddeaugrai O, Geater A (2016). Chikungunya and dengue virus infections during pregnancy: seroprevalence, seroincidence and maternal-fetal transmission, southern Thailand, 2009-2010. Epidemiol Infect.

[29] Cerny T, Schwarz M, Schwarz U, Lemant J, Gerardin P, Keller E (2017). The Range of Neurological Complications in Chikungunya Fever. Neurocrit Care.

[30] Inglis FM, Lee KM, Chiu KB, Purcell OM, Didier PJ, Russell-Lodrigue K (2016). Neuropathogenesis of Chikungunya infection: astrogliosis and innate immune activation. J Neuro-Oncol.

[31] Das T, Hoarau JJ, Jaffar Bandjee MC, Maquart M, Gasque P (2015). Multifaceted innate immune responses engaged by astrocytes, microglia and resident dendritic cells against Chikungunya neuroinfection. J Gen Virol.

[32] Chiam CW, Chan YF, Ong KC, Wong KT, Sam IC (2015). Neurovirulence comparison of chikungunya virus isolates of the Asian and east/central/south African genotypes from Malaysia. J Gen Virol..

[33] Verma R, Sahu R, Holla V (2014). Neurological manifestations of dengue infection: a review. J Neurol Sci.

[34] Li H, Saucedo-Cuevas L, Shresta S, Gleeson JG (2016). The neurobiology of Zika virus. Neuron..

[35] PAHO/WHO (2017). Pan American Health Organization / World Health Organization. Zika-Epidemiological Report Curacao.

[36] Smalley C, Erasmus JH, Chesson CB, Beasley DWC (2016). Status of research and development of vaccines for chikungunya. Vaccine..

