# Supplementary material for: Neurologic sequelae of severe chikungunya infection in the first 6 months of life: a prospective cohort study 24-months post-infection
Source: BMC Infect Dis. 2021 Feb 16;21:179. doi: 10.1186/s12879-021-05876-4 (PMC7885242; doi:10.1186/s12879-021-05876-4)
Supplement: Supplementary file 1 — Additional file 1. [file 12879_2021_5876_MOESM1_ESM.docx]

| **Birth - Admission** | | | | **Clinical diagnosis** | | | | **Laboratory diagnosis** | | | | | | |
| --- | --- | --- | --- | --- | --- | --- | --- | --- | --- | --- | --- | --- | --- | --- |
|  | Gestational age | Maternal ChikV symptoms | Age at admission | Fever | Rash | Rash localisation | Neurologic symptoms | Timing of sample | Child IgM | Child IgG | Timing of maternal sample^#^ | Maternal IgM | Maternal IgG | Diagnosis |
|  | (weeks/days ) | (days^$^) | (days) |  |  |  |  | (days*) | (g/L) | (g/L) | (days) | (g/L) | (g/L) |  |
| 1 | 38 / 6 | 1 | 0 | - | + | Full body | + | 23 | 7.66 | 4.11 | 6 | 5.64 | 0.14 | Child's IgM > 1 |
| 2 | 38 / 0 |  | 44 | + | - |  | + | 5 | 5.11 | 0.00 |  |  |  | Child's IgM > 1 |
| 3 | 37 / 2 |  | 184 | + | - |  | + | 5 | 3.10 | 0.36 |  |  |  | Child's IgM > 1 |
| 4 | 38 / 5 |  | 73 | + | + | Full body | + | 169 | 0.82 | 4.39 |  |  |  | Increase IgG > 4 |
| 5 | 40 / 0 |  | 17 | + | + | Full body | + | 32 | 7.69 | 4.17 |  |  |  | Child's IgM > 1 |
| 6 | 36 / 5 |  | 19 | + | + | Legs | + | 6 | 6.07 | 0.36 |  |  |  | Child's IgM > 1 |
| 7 | 38 / 5 |  | 113 | + | - |  | + | 5 | 5.64 | 0.53 |  |  |  | Child's IgM > 1 |
| 8 | 40 / 0 |  | 56 | + | + | Full body | + | 4 | 6.14 | 0.15 |  |  |  | Child's IgM > 1 |
| 9 | 39 / 0 |  | 132 | + | + | Legs with bullae | + | 10 | 8.56 | 2.10 |  |  |  | Child's IgM > 1 |
| 10 | 40 / 0 |  | 39 | + | + | Full body | + | 4 | 7.14 | 0.15 |  |  |  | Child's IgM > 1 |
| 11 | 39 / 3 | 0 | 7 | + | - |  | + / convulsion | 6 | 7.44 | 0.08 | 4 | 2.02 | 0.33 | Child's IgM > 1 |
| 12 | 35 / 0 |  | 149 | + | - |  | + | 203 | 1.40 | 3.90 |  |  |  | First sample missing  Child's IgM > 1 |
| 13 | 40 / 6 |  | 74 | + | + | Full body | + | 134 | 0.28 | 4.15 |  |  |  | First sample missing  Repeat IgG at 4 month post infection > 4 |
| 14 | 34 / 5 |  | 106 | + | + | Full body and bullae | + | 171 | 0.21 | 5.28 |  |  |  | First sample missing  Repeat IgG at 4 month post infection > 4 |
| 15 | 41 / 2 |  | 34 | + | + | Full body | + | 8 | 2.59 | 0.09 |  |  |  | Child's IgM > 1 |
| 16 | 40 / 0 |  | 83 | + | - |  | + | 162 | 0.56 | 4.20 |  |  |  | First sample missing  Repeat IgG at 4 month post infection > 4 |
| 17 | 37 / 2 | 1 | 0 | + | + | Full body | + | 11 | 0.73 | 0.04 | 7 | 5.53 | 0.04 | Mother's IgM > 1. Child's IgM > 0.5 |
| 18 | 40 / 0 |  | 37 | + | + | Full body | + | 3 | 1.31 | 0.14 |  |  |  | Child's IgM > 1 |

**Table S1.** Patient clinical and laboratory diagnosis

^$^ Number of days before birth that the mother’s initial ChikV symptoms started.

* Number of days after the start of the child’s initial symptoms of ChikV.

^#^ Number of days after the start of the mother’s initial symptoms of ChikV.
